# *Helicobacter pylori* Infection in Developing Countries: The Burden for How Long?

**DOI:** 10.4103/1319-3767.54743

**Published:** 2009-07

**Authors:** Barik A. Salih

**Affiliations:** Department of Biology, Fatih University, Faculty of Science, Istanbul, Turkey

**Keywords:** *Helicobacter pylori*, modes of transmission, preventative measures, prevalence of infection

## Abstract

Approximately 50% (over 3 billion) of the world populations are known to be infected with *Helicobacter pylori*, mainly in the developing countries. Among those, hundreds of millions of people develop peptic ulceration during their lifetime and still tens of millions might progress to gastric cancer. Possible modes of *H. pylori* transmission generally described are through direct contact between family members and also through contaminated water and food. Because the high prevalence of infection occurs mainly in developing countries and because the test-and-treat strategy puts a huge economic burden on many of these countries, it is time to take an immediate action toward this bacterial infection and adopt a strategy to prevent it. To address this issue, an updated prevalence of infection, modes of transmission, economics of infection and preventative measures to block the infection process have been discussed.

*Helicobacter pylori* infection is well known to be the most common human infection worldwide on the basis of the fact that approximately 50% of the world's populations are infected and that human beings are the main reservoir.[1,2] The pattern of infection is an early childhood acquisition of *H. pylori* (30%-50%) that reaches over 90% during adulthood in developing countries. This has been attributed to the poor socioeconomic status and overcrowded conditions.[3–5] Infection in developed countries is less common in young children and reaches up to 60% in older ages.[1,6,7] In the United States, a 20% infection rate among adolescents is being reported,[7] and recently an overall prevalence of 36% was reported, suggesting rapidly improving socioeconomic conditions.[8] While the prevalence of infection has decreased significantly in many parts of North America and Western Europe, no such decline has been noted in the majority of the developing world.[7]

*H. pylori* is the causative agent of chronic gastritis and peptic ulcer diseases and is an important risk factor for the development of gastric cancer and mucosal-associated lymphoid tissue (MALT) lymphoma.[9–11]

Although the world health organization (WHO) estimates indicate high infection rates among the world populations, most infected subjects develop no clinical symptoms or peptic ulceration and continue their life with superficial chronic gastritis.[12–15] However, still high percentages (approximately 17%) of the infected subjects will develop peptic ulcers and one-quarter of such patients (approximately 4.25%) even experience ulcer complications,[16] and still fewer (1%) will progress to gastric cancers.[10,16] If we consider these percentages in terms of numbers, then it is expected that several hundreds of millions (approximately 500 million) sooner or later will suffer from severe gastric pathology (peptic ulceration) and tens of millions (approximately 30 million) might progress to gastric cancer. A majority of these patients are within the populations of the developing countries.

## PREVALENCE OF INFECTION

All reports on the prevalence of infection indicate that subjects are infected with *H. pylori* early in childhood (<5 years of age) and that the risk of infection declines rapidly after that.[1–4,16,17] In developed countries [Table 1], Granstrom *et al*.[18] have monitored a cohort of Swedish children of age ranging from 6 months to 11 years and found that 13.6% were infected between 18 and 24 months of age; however, at age 11, only 3% of children were seropositive. Prevalence rates of 8.6%[19] and 2.4%[20] were reported for children aged 3 years in Ireland and in Germany, respectively. Recently, Okuda *et al*.[21] reported an overall rate of 3.7% in Japanese children aged <2 years. While in developing countries [Table 1] a prevalence rate of 22.6% was reported in Vietnamese children aged <3 years,[22] 46.7% in Mexican children <5 years,[23] 33% in Egyptian children <6 years,[24] 80% in Bangladeshi children <5 years,[25] 57% in Indian children <5 years,[26] 40% in Brazilian children <6 years,[27] 32.4% in Saudi children <10 years[28] and 41% in Turkish children <6 years.[29,30] Infection of subjects with *H. pylori* after this period continues but at much lower rate.[16] A seroconversion rate of 1.8% per year for children and 1.5% for adults was reported.[31] In another study, a reinfection rate of 2.0% per year was reported for children older than 5 years.[19] It was also shown that reinfection with *H. pylori* after treatment occurs rarely in children older than 5 years regardless of socioeconomic group or number of infected family members.[16] This might suggest that continuous contact between family members is required for the acquisition of *H. pylori* because children older than 5 years (school age) who spend less time at home are less commonly infected.

**Table 1:** Age-specific prevalence of *Helicobacter pylori* infection in children from various countries

Country	Age (years)	*H. pylori* infection (%)	Reference
Developed country			
Sweden	<2	13.6	15
Ireland	<3	8.6	14
Germany	<3	2.4	17
Japan	<2	3.7	18
Developing country			
Vietnam	<3	22.6	19
Mexico	<5	46.7	20
Egypt	<6	33.0	21
Bangladesh	<5	80.0	22
India	<5	57.0	23
Brazil	<6	40.0	24
Saudi Arabia	<10	32.4	25
Turkey	<6	41.0	26

In adults, the *H. pylori* infection rates are even higher and increase with age. A recent report from India indicates that almost 80% of the population is infected with *H. pylori.*[32] Similarly, Mishra *et al*.[26] indicated that earlier report showed 80–90% infection rates by the age of 20 years. In another recent report, the overall prevalence of *H. pylori* was detected to be 80% that reached 90% in subjects aged 70-79 years.[33] Considering the current population of India, which is 1.15 billion according to the United States Census Bureau,[34] around 918 million people (80%) are currently infected and the rates are not different from those reported in 1991. In China, an early report (1995) showed an overall 61% prevalence rate of *H. pylori* infection,[35] and in 2008, 2 reports showed similar percentages of 62%[36] and 60-70%,[37] indicating no changes in the prevalence of *H. pylori*, but on the contrary an increase in the infected population had occurred. The current population of China is 1.33 billion,[34] which also indicates that approximately 824 million people are infected. Reports from other highly populated developing countries showed similar infection rates. In Brazil, an overall prevalence rate of 65% was reported in healthy individuals.[38] In Bangladesh, *H. pylori* prevalence of more than 90% was reported in asymptomatic adults.[25] We have also found an overall 70% infection rate in asymptomatic Turkish subjects that reached up to 100% in subjects aged 60-69 years.[30]

Recently, a study on children aged 2-19 years in St. Petersburg, Russia, showed an overall decline in *H. pylori* prevalence rate from 44% in 1995 to 13% in 2005. The prevalence decreased from 30% among children younger than 5 years in 1995 to 2% after 10 years in the same age group. The authors correlated these findings with the improvements in the standards of living.[39] Similarly, reports from South Korea showed an overall *H. pylori* infection rate of 75% in adults in 1996,[40] 66.9% in 1998 that decreased to 59.6% in 2005[41] and, more recently,[42] a decline from 64.7% to 40.0% in the 8-year period (1998-2005) has been reported. Whether this drop is a local phenomenon or a national trend has to be investigated in those countries. However, there is a common consensus that the risk of acquisition and transmission of *H. pylori* can be minimized and prevented to a large extent by implementation of household hygiene, boiling water for drinking purposes and proper cleaning of vegetables.

## MODES OF TRANSMISSION

Several modes of transmission of *H. pylori* have been described in the literature; these included direct contact between subjects, which is considered the most common mode,[2,43–45] contaminated water sources and food[2,46–48] and, less commonly, iatrogenic transmission (during endoscopies and dental care)[2] and zoonotic transmission.[2,48]

### Direct contact

For several years now researchers have attempted to define the exact mode of transmission of *H. pylori* and the role of subjects in this process.

### Role of mother-to-child

Many investigators have studied the role of familial clustering in the spread of *H. pylori* infection and the majority indicated that mothers play the key role in transmission of *H. pylori* to their children and spouses.[43–45,49–58] In a study conducted on children of the Canadian First Nations community who were negative for *H. pylori* at entry, 16% acquired *H. pylori* over 1 year period from their positive mothers.[48] Malaty *et al*.[55] examined the longitudinal changes in *H. pylori* infection monitored from 1986-1994 within 46 families with children living in Japan. At study entry, prevalence of *H. pylori* in children with positive mothers was 23% versus 5% in children with negative mothers. Their data strongly support the key role of the infected mothers in the transmission within families and also indicated that seroconversion rate of 1.5% per year was evident only among children living with positive mothers that did not differ among adults living with or without children.

Tindberg *et al*.[49] compared the risk of *H. pylori* infection between family members and extraneous child-to-child transmission among Swedish school children with varying family backgrounds. They found 2% seroprevalence among children with Scandinavian parents and 55% among children with origin in high prevalence areas (Middle East and Africa). They concluded that intrafamilial transmission is far more important than child-to-child transmission outside the family. In a study from Eastern Turkey, it was shown that seroprevalence was also higher in children (49%) whose mothers were infected than in children (22.6%) of uninfected mothers.[51] Malaty[16] reported that if one index parent is positive for *H. pylori* then 40% of children were found to turn positive, while if both parents are negative only 3% of children turn positive. In China, it was also reported that in families with at least one infected parent, 85% of children were *H. pylori* positive, while in families with both parents uninfected, only 22% of children were *H. pylori* positive.[59] Similarly, investigators from Brazil reported that seropositivity was higher in family members of infected symptomatic index subjects than family members of uninfected index subject, especially mothers and siblings. They emphasized the role of mothers and siblings but not fathers in the transmission of *H. pylori.*[53] In a community-based cross-sectional study in 533 participants from 135 household members, with multiple generations living in the same house in Vietnam, *H. pylori* infection in children was found significantly associated with infection of the mothers, which supports the hypothesis of intrafamilial transmission.[60]

It appears that in developing countries, overcrowded conditions that create closer contacts between mothers and children and between siblings sharing the same bed might be the main reason for the high infection rates reported. In addition, in these countries working fathers had little contact with their children and that is why transmission of *H. pylori* was mostly linked to the mother.

### Role of spouse-to-spouse

In a recent study in Germany, prevalence of infection was 34.9% among women whose partners were infected and 14.5% when the partner was not infected.[61] Malaty[16] reported that if one spouse is positive for *H. pylori* then 68% of spouses turn positive, while if negative 9% of spouses were found positive. We also found in Turkey that *H. pylori* infection was significantly higher among married subjects and those with low socioeconomic status.[30] It was also reported that the risk of infection increases with the number of years lived with an infected partner. A 42% of subjects were found positive when living with an infected partner, while only 7% were infected when living with an uninfected partner.[62] These results support the hypothesis of a major role of spouse-to-spouse transmission of *H. pylori* infection and that continuous contact is required for the establishment of such infection.

### Transmission among non-family members

Triantafillidis *et al*.[63] investigated whether medical or non-medical staff in a large acute care hospital was at increased risk of acquiring *H. pylori* infection over a 5-year period. They observed an annual seroconversion rate of 4.95% and found that nursing staff had a significantly higher risk of infection compared with administrative and technical staff. The person-to-person mode of transmission is supported by the higher incidence of infection among institutionalized children and adults and the clustering of *H. pylori* infection within families. Also in support to this concept is the detection of *H. pylori* DNA in vomitus, saliva, dental plaque, gastric juice and feces.[2] The abovementioned data suggest that transmission of *H. pylori* can occur between individuals irrespective of their close relations provided that they maintain close contact for long periods.

### Contaminated water sources and food

Klein *et al*.[46] reported earlier that the municipal water supply seems to be an important source of *H. pylori* infection among Lima children from families of both low and high socioeconomic status. Consumption of uncooked vegetables has also been described as a mode for transmission of *H. pylori*.[47] Goodman *et al*.[48] also reported that children swimming in rivers or pools as did using streams as a drinking water source and those who frequently consumed raw vegetables were more likely to have the infection.

For the general population, it appears that the most likely mode of transmission is direct contact, by either the oral-oral route (through vomitus and saliva) or perhaps the fecal-oral route. Waterborne transmission, probably due to fecal contamination, may also be an important source of infection, especially in parts of the world in which untreated water is common.[2] Overall, inadequate sanitation practices, low socioeconomic status and overcrowded or high-density living conditions seem to be related to higher prevalence of *H. pylori* infection.[2] These studies suggest that the main critical period of life for acquiring *H. pylori* infections is the first 5 years of childhood and at adulthood were married couples living together.

## ECONOMICS OF *H PYLORI* INFECTION

Health care is becoming more and more expensive as accompanied by the increase in the standards of living. More importantly, the increase in population size mainly in developing countries makes coverage for health insurance a big burden on governments. The economics of *H. pylori* infection have been analyzed using the test and treat strategy and evidence for effectiveness gained from randomized controlled trials suggested that treating *H. pylori* is also more effective and less expensive than continuous PPI therapy, and is therefore the dominant strategy in treating peptic ulcer disease.[64]

Ladabaum *et al*.[65] have estimated the cost per dyspeptic patient managed by the test and treat strategy ($545) or by the PPI ($529) and recommended the first strategy. Delaney *et al*.[66] also found the test and treat strategy to be more effective than acid suppression alone and reported a mean additional cost of US$401 for endoscopy. Ebell *et al*.[67] have investigated the cost per quality-adjusted life year (QALY) and reported that two strategies are reasonable for patients presenting with dyspepsia: (1) empiric *H. pylori* eradication that costs $1198 per QALY, and (2) use of a serum *H. pylori* titer to identify patients who might benefit from *H. pylori* eradication at a cost of $1214 per QALY. In China, comparison of the incremental costs per ulcer treatment using the test and treat strategy, endoscopy and empirical PPI therapy showed US$1778, US$1797 and US$2158, respectively.[68] In Finland, Farkkila *et al*.[69] indicated that the test and treat strategy significantly reduced peptic ulcer disease and improved dyspeptic symptoms and quality of life but did not reduce the number of endoscopies. Mason *et al*.[70] in their randomized controlled trial using economic model suggested that population *H. pylori* screening and treatment for 1,000,000 45-year-olds would save over 6,000,000 pounds sterling and 1300 years of life. In another study, the cost-effectiveness of performing routine biopsies for the detection of *H. pylori* in non-ulcer dyspepsia patients showed an incremental cost of $10,716 per 100 treated patients.[71]

Most of these estimates came from studies conducted in developed countries and unfortunately no sufficient data is available yet from developing countries. Owing to the huge number of infected subjects in those countries, the number of routine upper endoscopies conducted is also very high. We have reported on such numbers in our recent study. On average 1500 endoscopies are being conducted in one year by a single clinician in a state hospital in Istanbul.[72]

From the abovementioned studies it is obvious that the health economics of managing *H. pylori* infection to prevent the occurrence of peptic ulcer and gastric cancer is highly expensive. With the enormous and continuous increase in the world population size, it is going to be very difficult to deal with such an infection even in the immediate future, particularly in developing countries. According to the United States Census Bureau[34] the world population will reach 7.5 billion in the year 2020, which indicates that an additional several hundred millions of infected subjects will be added to the already currently infected over 3 billion subjects. Such an increase will have an even greater impact on the economic burden that is already being overloaded. With all evidence being pointed toward the familial clustering of *H. pylori,* the main focus then is to block the process among family members and improve sanitary measures.

## PREVENTATIVE MEASURES

Establish a campaign for educating people, particularly infected mothers and newlywed couples about familial clustering of *H. pylori* infection and the risk of infecting others by direct contact and exchange of saliva.

Implementation of sanitary measures among family members (hand and mouth wash, brushing teeth, no sharing of food plates or drinking glasses, no sharing of spoons in feeding children, no bed sharing between siblings).

Routine testing of newlywed couples (depends on the availability of equipments and cost of the test). If they are found to be *H. pylori* positive, recommendations should be provided for strict implementation of sanitary measures to prevent partner infection and also future infection of their children. Marshall *et al*.[73] have indicated earlier that in low-risk asymptomatic patients with dyspepsia, testing for *H. pylori* using serology appears to be economical and that ^13^C-labeled urea breath test (^13^C-UBT) may also be a cost-effective alternative to serology depending on the current cost of each test. In a study on patient acceptance of tests, it was reported that the stool test would be less popular than a blood test or UBT and may have less compliance. Patients might not come back to bring the stool specimen; however, the blood test or UBT can be done before they leave.[74]

Recommendations for adopting the ^13^C-UBT test as an additional mandatory test together with the already existing mandatory tests (HIV, HBV, HCV and Syphilis) required for all newlywed couples before registration. This will help partners in applying strict sanitary measures so as to not transfer infection between each other and to their future children and also in future follow-up of subjects who develop dyspepsia for treatment recommendation.

Test-and-treat strategy for mothers with recurrent dyspepsia; if found *H. pylori* positive, a recommendation for testing family members.

Routine testing of drinking water sources for *H. pylori* contamination, especially in poor communities and recommendations for boiling water before drinking.

The issue of vaccination and the status of vaccine against *H. pylori* is currently still under development. According to Kabir[75] who indicated that although extensive studies in the mouse model have demonstrated the feasibility of both therapeutic and prophylactic immunizations, the mechanism of vaccine-induced protection is poorly understood as several factors such as immunoglobulin and various cytokines do not contribute to protection. Svennerholm *et al*.[76] also reported that there is still a strong need to clarify the main protective immune mechanisms against *H. pylori* and to identify a cocktail of strong protective antigens, or recombinant bacterial strains that express antigens that could be administered by a regimen that gives rise to effective immune responses in humans.

## CONCLUSIONS

Because most *H. pylori* infections acquired during early childhood, particularly in children aged less than 5 years, a continuous contact is required for the establishment of a real infection that can last lifelong. Infection rates are lower after this period due to the fact that less contact occurs between mothers and children. This is because children start their school year and spent more time outside their home. Infection with *H. pylori* continues to be acquired by children after that but at lower rates depending on the mode of transmission. During adult life, however, married couples are also at high risk of infection if one spouse is infected. Keeping this in mind, serious measures should be taken immediately to advocate the implementation of sanitary conditions and education against the transmission of *H. pylori* to block the infection process.
